# Contact Lens Health Week — August 21–25, 2017

**DOI:** 10.15585/mmwr.mm6632a1

**Published:** 2017-08-18

August 21–25, 2017, marks the fourth annual Contact Lens Health Week. In collaboration with partners from the clinical, public health, industry, and regulatory sectors, CDC is promoting healthy wear and care practices to reduce the risk for eye infections among the approximately 45 million persons in the United States who wear contact lenses. Research after outbreaks of rare but serious eye infections in the United States has indicated that these infections occur most often in contact lens wearers who do not take proper care of their contact lenses, indicating a need to promote safer wear and care (*1*,*2*).

A report in this issue of *MMWR* describes CDC’s first-ever population-based estimates of contact lens–related risk behaviors in persons aged 12–17 years (referred to here as adolescents) in the United States. Approximately six in seven adolescents reported at least one behavior (e.g., sleeping in lenses, swimming, or not replacing lenses and storage cases as recommended) putting them at risk for a serious contact lens–related eye infection. Encouraging adolescents to adopt healthy contact lens wear and care habits might help them maintain healthy habits into adulthood.

Although most contact lens wearers receive the benefits of vision correction, contact lenses can pose an infection risk, especially if they are not worn and cared for properly. Practicing proper contact lens hygiene and regularly visiting an eye care provider are important behaviors for keeping contact lens wearers’ eyes healthy. Additional information on Contact Lens Health Week and the proper wear and care of contact lenses is available at https://www.cdc.gov/contactlenses.
